# Sero-Surveillance to Assess Immunity to Rubella and Assessment of Immunogenicity and Safety of a Single dose of Rubella Vaccine in School Girls

**DOI:** 10.4103/0970-0218.62575

**Published:** 2010-01

**Authors:** Hitt Sharma, Sunil Chowdhari, Tilak Raj Raina, Subodh Bhardwaj, Gajanan Namjoshi, Sameer Parekh

**Affiliations:** Serum Institute of India Ltd, Pune, India; 1 Government Medical College and Associated Hospitals, Jammu, India; 2 Earlier with Serum Institute of India Ltd, Pune, India

**Keywords:** Immunogenicity, Rubella, school girls, serosurveillance, India

## Abstract

**Background:**

Rubella vaccination is not yet included in National Immunization Schedule in India. Serosurvey is frequently used to assess epidemiologic pattern of Rubella in a community. Serosurveys in different parts of India have found that 6–47% of women are susceptible for Rubella infection. The present serosurveillance was conducted in Jammu, India, in two public schools.

**Objective:**

To determine serological status of Rubella antibodies of school girls and assessment of immunogenicity and reactogenicity of Rubella immunization in seronegative girls.

**Materials and Methods:**

The current study was conducted to determine Rubella serostatus in peripubertal schoolgirls aged 11–18 years and also to assess immunogenicity and safety of Rubella vaccine (R-Vac) of Serum Institute of India Ltd., Pune, in seronegative girls. For screening, pre-vaccination serum Rubella IgG antibodies were determined and to assess immunogenicity of the vaccine, post-vaccination IgG antibodies were compared with pre-vaccination levels. Safety assessment was done for a period of 8 weeks, post-vaccination.

**Results:**

A total of 90 (32.7%) seronegative girls were vaccinated. All girls (100%) became seropositive, post-vaccination. Clinically relevant and statistically significant increase in anti-Rubella IgG titres was observed. The adverse events were mild and self-limiting.

**Conclusions:**

R-Vac vaccine used in the study demonstrated an excellent safety and immunogenicity profile.

## Introduction

Rubella occurs worldwide and is normally a mild childhood disease. However, infection during early pregnancy may cause fetal death or Congenital Rubella Syndrome (CRS). CRS can lead to growth retardation, eye defects, deafness, cardiac defects, microcephaly, mental retardation, hepatitis, bone lesions, interstitial pneumonitis, diabetes mellitus and psychiatric disorders.(1) Infants with CRS shed Rubella virus for long periods in nasopharyngeal secretions and urine and transmit Rubella infection to close contacts.

CRS is an important cause of hearing and visual impairment and also mental retardation, in countries where acquired Rubella infection has not been controlled or eliminated. Reliable statistics on CRS are rare in developing countries. WHO estimates that worldwide more than 100 000 children are born with CRS each year, and most of them in developing countries.(2)

Rubella is a vaccine preventable disease and the primary purpose of Rubella vaccination is to prevent the occurrence of congenital Rubella infections. Following well-designed and implemented programs, Rubella and CRS have almost disappeared from many countries.(3) Rubella is not a notifiable disease in many countries and its clinical diagnosis is frequently inaccurate. Serosurveys are therefore used to assess the epidemiologic pattern of Rubella in a community. In India, a few surveys have been conducted to detect Rubella IgG seropositivity status in women of childbearing age. From these surveys it is observed that seropositivity in this population is from 53 to 94.1%. Thus, remaining women are susceptible to Rubella and potentially at risk of infection during pregnancy.(4–8)

For updating the information available, another such survey was conducted in the year 2000, in Jammu region. As earlier, surveillance was never done in that region, it was therefore considered worthwhile to study the Rubella seroprevalence rates and also to assess immunogenicity and safety of R-Vac in peripubertal seronegative schoolgirls.

## Materials and Methods

The study was undertaken with the objective of conducting Rubella serosurveillance and assessing the safety and immunogenicity of R-Vac in seronegative girls. Sample size comprised of total 275 girls aged 11–18 years. Two public schools from Jammu region, India, participated in the study. The study was conducted following Good Clinical Practice guidelines and Declaration of Helsinki.

### Ethical clearance

The study was permitted by Institutional Ethics Committee of Government Medical College and associated Hospitals, Jammu. The parents of schoolgirls were informed about the study and written informed consent was obtained from all the parents for their inclusion in the study. Principals of these schools also gave informed consent for participation of the students in the study.

### Inclusion and exclusion criteria

Inclusion criteria included peripubertal school girls aged 11–18 years, not suffering from any illness or any immunological disorder, not participating in any other clinical trial and whose parents were willing to give written informed consent. Exclusion criteria included girls with known Rubella infection or vaccination, pregnancy, corticosteroid therapy, immunosuppressive therapy, radiotherapy, leukemia, acute infectious disease, renal or cardiac dysfunction, gamma globulin or blood transfusion.

### Vaccine and dosage

Live-attenuated R-Vac (lyophilized) is manufactured by Serum Institute of India Ltd., Pune, using Wistar RA 27/3 strain of the virus. Rubella vaccine virus is propagated on Human Diploid Cell. The vaccine is reconstituted with water for injection and each 0.5 ml dose contains not less than 1000 CCID_50_ of live-attenuated virus. The vaccine meets the requirements of WHO when tested by the methods outlined in WHO TRS 840 (1994). R-Vac is commercially available and registered in India. A 0.5 ml single dose of vaccine was administered subcutaneously into the upper arm.

### Serology

Pre-vaccination (2 ml) venous blood from 275 enrolled schoolgirls was drawn and collected in vacutainers with gel separators, serum was separated and stored at a temperature of < −20° C, before testing. Out of the total schoolgirls screened, 90 (32.7%) girls were found to be seronegative for Rubella IgG who received R-Vac. Post-vaccination blood sample was collected 8 weeks after vaccination.

Sera samples were tested for Rubella IgG antibodies, using commercially available Enzyme-Linked Immuno Sorbent Assay (ELISA) kits from Novum Diagnostica, Germany. Serology was conducted at the Department of Blood Transfusion Medicine, SMGS Hospital, Government Medical College, Jammu. Good Laboratory Practices were followed while conducting the tests. The cut-off values used as per the kit literature were: Rubella IgG: <15 IU/ml: Negative, 15 to <25 IU/ml: Indeterminate and ≥ 25 IU/ml: Positive.

### Safety assessment

Following vaccination, all girls were asked to wait and monitored for first 15 min to detect and treat any immediate allergic reaction or anaphylaxis. Thereafter, safety of R-Vac was evaluated actively by following up the vaccinated schoolgirls, daily for first 3 days and then once a week for 8 weeks after vaccination. In addition, solicited local adverse events were pain, redness, induration, swelling and abscess. Solicited systemic adverse events were fever, rash, arthritis, arthralgia, thrombocytopenia, neuropathies, anaphylaxis, headache, sore throat, lymph node enlargement, convulsions and changes in menstruation.

## Results

A total of 275 girls with mean age 14.04 (± 0.32) years eligible as per inclusion and exclusion criteria were enrolled in the study. On screening, 185 (67.3%) girls were found to be seropositive and 90 (32.7%) girls negative, for IgG Rubella antibodies.

### Immunogenicity

**Figure 1:**
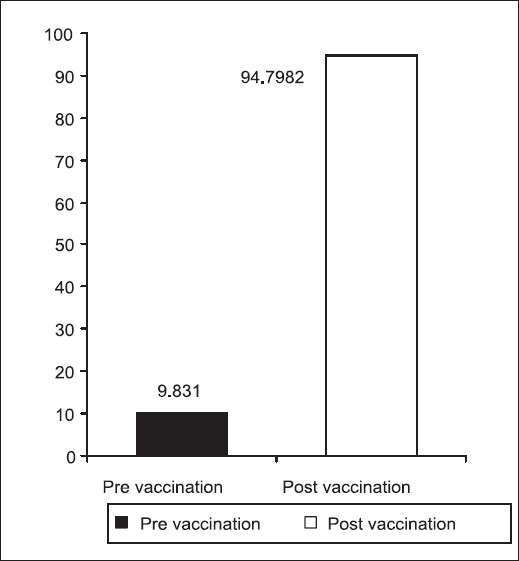
IgG Rubella antibody titres (GMT) (IU/ml)

**Figure 2:**
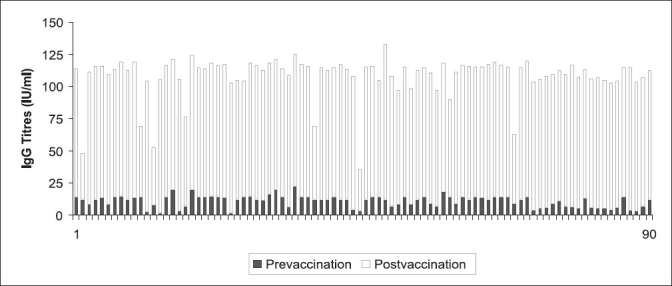
Pre- and post-vaccination IgG titres of 90 seronegative girls

Pre-vaccination, IgG Rubella antibody was 9.83 IU/ml, which increased to 94.8 IU/ml after 8 weeks, following vaccination [Figure 1]. There was a clinically relevant and statistically highly significant difference (*P*<0.01, Wilcoxon's Signed Rank Test) between the pre- and post-vaccination Rubella IgG GMTs. Post-vaccination, all seronegative girls attained Rubella IgG antibody titre above the protective cut-off of 25 IU/ml, leading to 100% seropositivity [Figure 2].

### Safety

A total of 5 (5.5%) girls experienced pain and swelling at injection site and 3 (3.3%) girls complained of mild fever (<100° F). Both these adverse events resolved, without any medication. Two girls (2.2%) complained of irregular menstrual period, which was unrelated to vaccination. No other adverse event was reported. No serious adverse event like anaphylaxis or hospitalization was observed during the study. Rubella vaccine was found to have an excellent tolerability profile.

## Discussion

Rubella is a mild exanthematous illness, but infections during the first 3-4 months of pregnancy can result in spontaneous abortion, still birth and CRS.(9,10) There is a considerable variation in the prevalence of Rubella antibodies among women of childbearing age. The presence of Rubella-specific IgG in an unvaccinated population is a long-term marker of previous Rubella infection. These IgG antibodies persist for long term and protect the individual from Rubella infection.(11,12)

The current study was planned to assess the seroprevalence of Rubella IgG antibodies as the titres below the cut-off value are associated with a risk of acquiring Rubella infection. The population chosen was peripubertal schoolgirls as the girls and women of this age group should be targeted when aim is to prevent CRS.

The results of our study revealed that out of the total 275 girls, 185 (67.3%) were seropositive and 90 (32.7%) were seronegative for IgG Rubella antibodies. In a study at Amritsar, India, which is near Jammu, it was observed that 64% girls in pre-pubertal age group of 10–15 years were Rubella IgG seropositive.(13) Seroprevalence and incidence of Rubella in and around New Delhi, India, conducted by National Institute of Communicable Diseases (NICD), New Delhi, substantiated the fact that though immunity status against Rubella in women of childbearing age group increased steadily from 1988 to 2002, approximately 10 to 15% of women reached childbearing age without developing immunity against Rubella virus and were at high risk of contracting infection during pregnancy.(14)

Another serosurvey in New Delhi conducted on adolescent girls in the age group of 15–18 years found that overall Rubella IgG seronegativity was 17.83%.(15)

Our results are in accordance with earlier studies and highlight the importance of serosurveillance and vaccination of subjects in the susceptible cohort. A statistically significant rise in Rubella IgG titres was observed with 100% seropositivity, post-vaccination. Rubella vaccine (R-Vac) was tolerated well and the adverse events observed were mild and self-limiting. It is evident from the results that there is a definite need to protect the at risk group from Rubella infection, and Rubella vaccination is the ultimate choice, which can serve the purpose. Looking at sero-surveys and incidence of Rubella in India, like many other countries, efforts should be made to introduce the first dose of Rubella containing vaccine on a large scale, during the second year of life. This can greatly reduce the number of women at risk of infection during pregnancy, thereby controlling the incidence of CRS.
